# Clinical Utility of Rapid-Sequence Magnetic Resonance Imaging (rMRI) in Pediatric Neurosurgery: Enhancing Assessment of Hydrocephalic Changes †

**DOI:** 10.3390/children13050699

**Published:** 2026-05-20

**Authors:** Johanna Krämer, Nieke Ueding, Christian Ott, Angelika Seitz, Malte Ottenhausen, Sandro M. Krieg, Ahmed El Damaty, Mohammed Issa

**Affiliations:** 1 Department of Neurosurgery, Hospital University Heidelberg, Im Neuenheimer Feld 400, 69120 Heidelberg, Germany; 2 Faculty of Medicine, Heidelberg University, 69120 Heidelberg, Germany; 3 Department of Neuroradiology, Hospital University Heidelberg, 69120 Heidelberg, Germany

**Keywords:** pediatric neuroimaging, hydrocephalus, pediatric brain tumor, rapid-sequence MRI

## Abstract

**Highlights:**

- Rapid-sequence MRI enabled clinical decision-making in 37.3% of emergency cases, including surgery and shunt adjustments.
- rMRI showed high feasibility with minimal sedation (5.1%) and rare need for follow-up CT (1.9%).
- rMRI provides a safe, radiation-free alternative to CT for pediatric emergency neuroimaging.
- Implementation of rMRI protocols may reduce radiation exposure, particularly in children requiring repeated imaging (e.g., tumor surveillance).

**Abstract:**

**Objective**: Rapid-sequence magnetic resonance imaging (rMRI) has emerged as a radiation-free alternative to computed tomography (CT) in pediatric neuroimaging. This study aimed to evaluate the diagnostic utility, clinical consequences, and subgroup-specific differences in pediatric patients undergoing rMRI for emergency neurosurgical indications. **Methods**: We conducted a retrospective, single-center study of 158 rMRIs in 73 pediatric patients who underwent rMRI between January 2017 and December 2023 due to suspected complications of hydrocephalus and brain tumors. A short MRI protocol (195 s) including FLAIR BLADE and T2 HATSE sequences was employed. Patients were categorized into tumor non-related and tumor-related hydrocephalus groups. **Results**: A total of 158 rMRIs in 73 pediatric patients were included. The mean age was 7.09 ± 4.63 years, with 69.6% male patients. Ventricular size monitoring was the most common rMRI indication (96.2%). Clinical consequences followed in 52.5% of cases, including surgical interventions (21.5%) and shunt valve adjustments (37.3%). Sedation was required in only 5.1% of patients. Only 1.9% required follow-up CT. Statistically significant intergroup differences were found in drainage type (***p* < 0.001**), diagnosis (***p* < 0.001**), and need for follow-up MRI (17.4% in the tumor-related hydrocephalus group vs. 4.5% in the tumor non-related hydrocephalus group; ***p* = 0.014**). **Conclusions**: rMRI is a safe, efficient, and effective alternative to CT for emergency neuroimaging in pediatric patients with hydrocephalus, tumors, or shunts. It facilitates timely clinical decision-making while avoiding radiation exposure. Our findings support broader implementation of rMRI protocols, particularly for patients requiring ongoing tumor surveillance.

## 1. Introduction

Hydrocephalus and intracranial tumors are critical conditions in pediatric patients that require accurate and timely diagnosis to guide treatment [1,2]. Traditionally, computed tomography (CT) has been the primary imaging modality used in acute settings due to its rapid acquisition time and wide availability [3]. However, recent studies have demonstrated a linear dose–response relationship between CT-related radiation exposure and the development of brain tumors in children and adolescents [4]. Furthermore, estimates of CT utilization in the United States in 2023 indicate that, if current radiation dose levels and examination frequencies persist, CT-associated malignancies could, in the long term, account for up to 5% of all newly diagnosed neoplasms annually [5].

However, concerns about radiation exposure in children have led to an increasing interest in alternative imaging techniques, particularly magnetic resonance imaging (MRI) [2,6]. In recent years, rapid-sequence MRI (rMRI) has emerged as a potential alternative to CT, offering comparable diagnostic accuracy without the associated ionizing radiation [7,8]. rMRI utilizes shortened imaging protocols that can be performed in significantly less time than conventional MRI, often eliminating the need for sedation in pediatric patients [6].

Several studies have demonstrated the efficacy of rMRI in pediatric neuroimaging. It has been shown to reduce the reliance on CT in emergency departments and to provide high sensitivity and specificity in detecting a variety of intracranial conditions, including hemorrhages, tumor brain injuries, and ischemic events [7,9,10,11,12,13,14]. Despite these advantages, challenges remain, such as the higher rates of unsuccessful imaging due to patient motion or hardware artifacts, as well as the longer acquisition times compared to CT [3,7]. Nonetheless, rMRI represents a significant advancement in pediatric neuroimaging, balancing the need for rapid diagnosis with the imperative of minimizing radiation exposure in vulnerable populations [7,14].

This innovative study aims to evaluate the efficacy and safety of rMRI in the assessment of hydrocephalus and intracranial tumors in pediatric patients, comparing its performance to traditional imaging modalities and exploring its potential to enhance clinical decision-making in this critical patient group.

### 1.1. Methods

Our single-center retrospective study included pediatric patients who underwent rMRI for neurosurgical indications between January 2017 and December 2023. The study adhered to the ethical principles outlined in the Declaration of Helsinki and received approval from the institutional Ethics Committee (Nr. S-084/2022). Informed consent was obtained from all patients or their parents/legal guardians.

The primary aim of the study was to evaluate the clinical utility and consequences of rMRI in various pediatric neurosurgical subgroups, including patients with tumors, cerebrospinal fluid (CSF) shunts, and those with both conditions. Brain imaging with rMRI was performed as part of diagnostic evaluation, postoperative follow-up, or surveillance of ventricular size and shunt function.

A comprehensive review of clinical records and imaging data was conducted, including variables such as age, gender, diagnosis, indication for rMRI, patient type (inpatient vs. outpatient), and the need for sedation. We further analyzed consequences following rMRI, including surgical interventions, shunt valve adjustments, and the requirement for additional imaging such as standard MRI or cranial CT. Hospital stay duration and follow-up intervals were also recorded.

**Figure 1:**
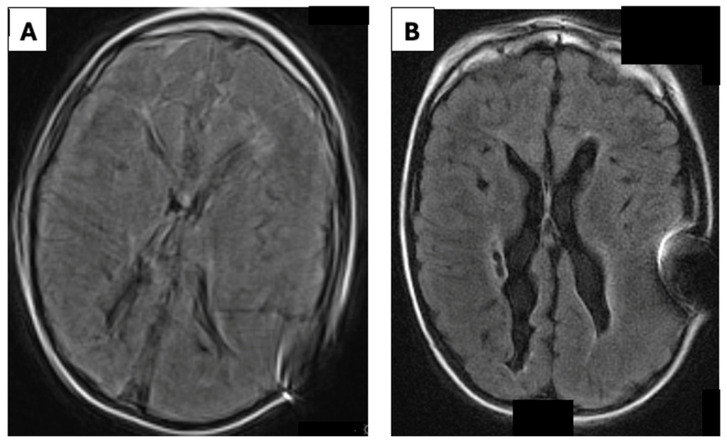
Rapid MRI (**A**) with axial T2-weighted fluid-attenuated inversion recovery (T2-FLAIR) sequences showing persistent overdrainage in an 8-year-old boy despite multiple valve adjustments. The consequence of this imaging is the surgical replacement of the defective valve. The postoperative Rapid MRI control (**B**) with T2-FLAIR showing the normalization of the ventricular size.

**Figure 2:**
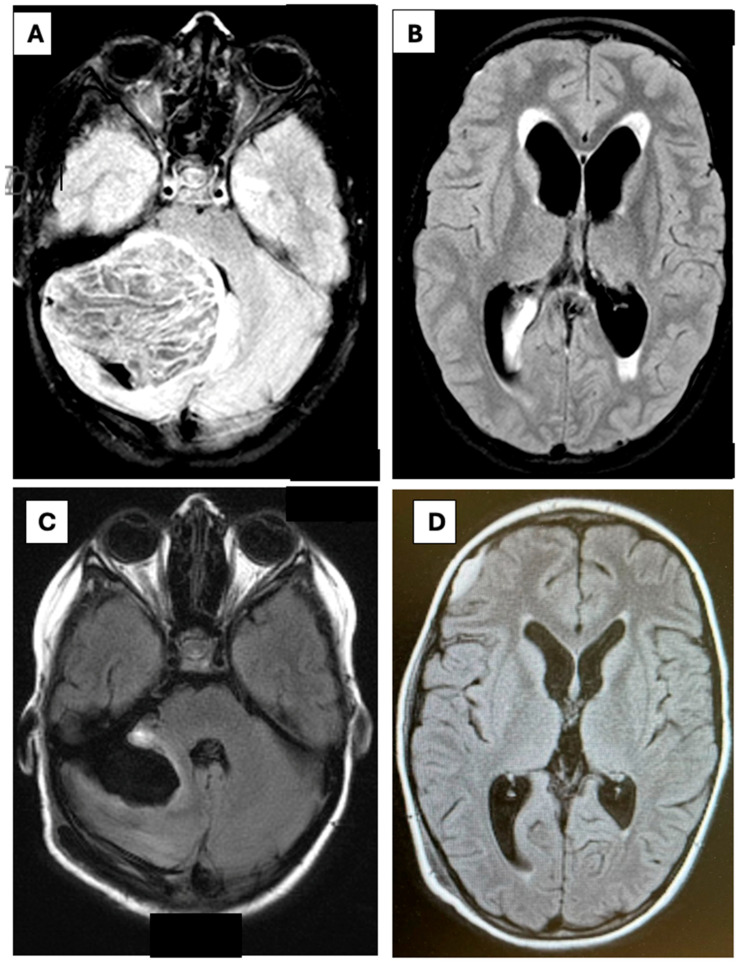
(**A**) Axial T2-weighted fluid-attenuated inversion recovery (T2-FLAIR) MRI demonstrating a cerebellar dysplastic gangliocytoma in a 10-year-old patient diagnosed with Lhermitte–Duclos Syndrome. (**B**) The same axial T2-FLAIR sequence reveals hydrocephalic enlargement of the ventricular system. (**C**) A follow-up rapid MRI using T2-FLAIR fluid-attenuated inversion recovery was performed 48 h after external ventricular drain (EVD) closure, prior to removal. Imaging shows re-expansion of the fourth ventricle to normal dimensions, indicating resolution of the mass effect previously exerted by the tumor. (**D**) Postoperative rapid MRI using T2-FLAIR imaging demonstrates reduced ventricular size and cerebrospinal fluid diapedesis, consistent with resolving hydrocephalus.

Patients were categorized into two subgroups (tumor non-related [Figure 1] and tumor-related hydrocephalus [Figure 2]) to allow for intragroup comparisons of clinical characteristics, diagnostic indications, and imaging-related outcomes. This classification was made to distinguish hydrocephalus caused by obstructive or compressive mass effects from those with non-tumoral etiologies, which may differ in pathophysiology, urgency, and treatment approach. The clinical impact of rMRI findings was assessed based on subsequent management decisions, including surgery or shunt-related interventions.

### 1.2. Imaging Protocol

All rapid-sequence MRIs (rMRI) were performed on a 1.5-T MRI system (MAGNETOM Avanto fit with BioMatrix, Siemens) using a standardized protocol optimized for speed and diagnostic efficiency. The rMRI protocol included the following sequences:

- *AAHead-Scout and AAHead-Scout-MPR tra:* These initial localizer sequences provided rapid anatomical orientation and ensured accurate positioning for subsequent imaging. They enabled multiplanar reconstructions to confirm the alignment of the imaging planes.
- ***FLAIR BLADE (transverse):*** A motion-robust T2-weighted fluid-attenuated inversion recovery sequence using BLADE (radial k-space acquisition by Siemens). This sequence provides high sensitivity for detecting periventricular fluid collections and parenchymal abnormalities while effectively suppressing CSF signal. It is particularly useful for identifying postoperative changes, such as hygromas or residual edema, and is well-suited for pediatric patients due to its reduced motion sensitivity.
- ***T2 HASTE (transverse):*** A single-shot, ultra-fast T2-weighted sequence (Half-Fourier Acquisition Single-shot Turbo spin Echo), used primarily to localize ventricular catheters and assess ventricular size. Its high speed and motion insensitivity make it ideal for uncooperative or unsedated pediatric patients.

The rapid MRI sequences used in this study were specifically optimized for structural assessment and ventricular evaluation. They were not designed to acquire hemodynamic, diffusion tensor imaging (DTI), or functional MRI (fMRI) data, as these techniques require substantially longer acquisition times and were beyond the scope of this rapid assessment protocol. Accordingly, the primary aim of rMRI in this setting was the rapid evaluation of hydrocephalus progression and ventricular changes. At our institution, rapid-sequence MRI has been the standard first-line imaging modality for pediatric hydrocephalus patients for several years. Therefore, CT scans were not routinely performed and were reserved only for cases in which rMRI findings were insufficient for further diagnostic clarification or therapeutic decision-making. rMRI was not preferentially reserved for particularly stable or cooperative patients. In younger or less cooperative children, motion reduction was frequently achieved with parental assistance by gently stabilizing the child’s head during image acquisition, thereby avoiding the need for sedation in many cases. Compared with CT, rapid MRI protocols using T2-weighted and FLAIR-BLADE sequences provide excellent soft-tissue contrast and precise assessment of ventricular size without exposing pediatric patients to ionizing radiation. Although CT remains faster and is commonly used in emergency settings, the implemented rMRI protocol allowed efficient evaluation of hydrocephalus progression and CSF space changes in a safe and radiation-free manner.

The total imaging time for the entire rMRI protocol was approximately 195 s, allowing for a fast yet comprehensive assessment suitable for pediatric patients, often without the need for sedation.

### 1.3. Statistical Analysis

Normal distribution of continuous variables was evaluated using the Shapiro–Wilk test. Data were expressed as mean ± standard deviation for continuous variables and as frequencies and percentages for categorical variables. Comparisons of continuous variables among the three study groups were performed using analysis of variance (ANOVA), while categorical variables were compared using the Chi-square test or Fisher’s exact test, as appropriate. A *p*-value of less than 0.05 was considered statistically significant. Statistical analyses were performed using SPSS 29 (IBM Corp., Armonk, NY, USA).

## 2. Results

### 2.1. Patients’ Characteristics

While demographic and disease-related characteristics are presented on a per-patient basis (*n = 73*), all imaging-related findings and clinical workflow data are reported per rMRI examination (*n = 158*). A total of 158 rMRIs in 73 pediatric patients were included in the study, comprising 47 males (64.4%) and 26 females (35.6%). The mean age at the time of imaging was 7.09 ± 4.63 years (range: 0.1–21.5 years). The mean follow-up duration was 21.92 ± 29.35 months. Patients were categorized into two study groups: those with tumor non-related hydrocephalus (52.1%) and those with tumor-related hydrocephalus (47.9%). Regarding cerebrospinal fluid (CSF) drainage, 87.7% of patients had internal drainage, 4.1% had external drainage, and 8.2% had no drainage at all. The most common diagnoses were congenital/post-hemorrhagic hydrocephalus (32.9%), followed by other conditions (%), medulloblastoma (12.3%), pilocytic astrocytoma (9.6%), and idiopathic aqueduct stenosis (5.5%).

The primary indication for rapid MRI (rMRI) was ventricular size monitoring, accounting for 96.2% of cases. Other indications included exclusion of hemorrhage (0.6%), exclusion of increased intracranial pressure (1.3%), postoperative follow-up (0.6%), shunt follow-up (0.6%), and evaluation of fluid in the mastoid cells (0.6%).

Most patients were inpatients (75.3%), while 24.7% were outpatients. Clinical consequences following rMRI were observed in 52.5% of cases, including surgical intervention in 21.5% and shunt valve adjustments in 37.3%. Sedation during rMRI was required in 5.1% of patients. Regular MRI follow-up after rMRI was indicated in 10.1% of cases, while 1.9% required regular cranial CT follow-up. (Table 1).

### 2.2. Intragroup Comparison

In a subgroup analysis comparing rMRI examinations performed in patients with tumor non-related hydrocephalus (n = 89) and those with tumor-related hydrocephalus (n = 69), statistically significant differences were observed in age (mean age: 6.3 ± 4.4 vs. 8.1 ± 4.7 years; *p* = **0.010**). Gender distribution also showed no significant difference (64.0% male in the tumor non-related hydrocephalus group vs. 76.8% in the tumor-related hydrocephalus group; *p* = 0.116). Diagnoses differed significantly between groups (***p* < 0.001**). Post-hemorrhagic hydrocephalus (55.1%), idiopathic aqueduct stenosis (11.2%), and congenital hydrocephalus (10.1%) were observed exclusively in the tumor non-related hydrocephalus group, while pilocytic astrocytoma (33.3%) and medulloblastoma (24.6%) were exclusive or more prevalent in the tumor-related hydrocephalus group.

CSF drainage methods also differed significantly (***p* < 0.001**). All cases in the tumor non-related hydrocephalus group had internal drainage (100%) with VP Shunts, whereas the tumor-related hydrocephalus group showed a broader distribution with internal (82.6%) with VP Shunts, external (7.2%) with external ventricular drain nor lumbar drain, and no drainage (10.1%). The most common indication for rMRI in both groups was ventricular size assessment (98.9% in tumor non-related hydrocephalus vs. 92.8% in tumor-related hydrocephalus; *p* = 0.259), with no significant differences in other indications.

While most patients in both groups were inpatients, the tumor-related hydrocephalus group had a higher proportion (82.6% vs. 69.7%; *p* = 0.066), though this was not statistically significant. Clinical consequences following rMRI, such as surgical interventions (56.2% vs. 42.7%), shunt valve adjustments (19.1% vs. 24.6%), and other actions, showed no significant differences (*p* > 0.1 for all). Sedation during rMRI was comparably rare in both groups (4.5% vs. 5.8%; *p* = 0.730).

However, the need for additional MRI follow-up was significantly higher in the tumor-related hydrocephalus group (17.4%) compared to the tumor non-related hydrocephalus group (4.5%) (*p* = **0.014**). Indications for follow-up MRI also varied significantly between groups (*p* = **0.027**), with staging, postoperative control, and preoperative planning more common in the tumor-related hydrocephalus group. Consequences resulting from follow-up MRI—such as shunt adjustments, operative revisions, or new shunt placements—were rare in both groups and did not differ significantly (*p* = 0.251) (see Table 2).

## 3. Discussion

This study confirms that rapid-sequence MRI (rMRI) is a feasible, safe, and diagnostically robust imaging tool in pediatric neurosurgical emergencies. Using a fast, motion-tolerant protocol—completed in approximately 195 s—we successfully evaluated ventricular size, shunt function, and tumor-associated pathology across a diverse patient population. Most importantly, the protocol allowed for reliable neuroimaging without ionizing radiation, even in acute clinical scenarios.

While much of the existing literature on rMRI focuses on acute traumatic or vascular events—including traumatic brain injury (TBI), intracranial hemorrhage, ischemic stroke, and neonatal seizures—our study expands the clinical scope of rMRI by applying it successfully to non-traumatic neurosurgical emergencies [6,8,10,11,12,13,14,15,16]. These include patients with hydrocephalus, tumor-related mass effects, or acute changes in shunt function. Despite this distinction, our findings are well aligned with prior research supporting rMRI as an alternative to CT in emergency settings. Despite advances in MRI technology, CT imaging remains a key diagnostic tool in the emergency assessment of complications in patients with hydrocephalus and intracranial tumors [1]. However, new studies associated a significant dose–response increase in hematologic malignancy risk in children and adolescents, with approximately 10% of hematological malignancies linked to imaging-related radiation especially CT [17]. Few earlier studies have mentioned the use of rapid MRI (rMRI) for the evaluation of ventricular width in the context of hydrocephalus, demonstrating comparable outcomes to those reported in the present study [18,19,20,21].

In addition to evaluating overall utility, among 158 rMRIs, 56.3% had CSF shunt-related pathology and 43.7% had combined tumor and hydrocephalus conditions. This classification was made to distinguish hydrocephalus caused by obstructive or compressive mass effects from those with non-tumoral etiologies, which may differ in pathophysiology, urgency, and treatment approach. Internal drainage was universal in the tumor non-related hydrocephalus group but more varied in the “tumor related hydrocephalus” group, which included external (7.2%) and no drainage (10.1%) (*p* < **0.001**).

Ventricular size monitoring was the leading rMRI indication across both groups (98.9% vs. 92.8%; *p* = 0.259), with sedation required in just 5.1% overall, because sedation time is intentionally kept to the absolute minimum. In these ~5% of cases, sedation is used briefly to obtain only the clinically indicated rMRI sequences; extending to a full MRI would unnecessarily prolong sedation and exposure without added clinical benefit. rMRI findings influenced management in 52.5% of cases, prompting surgical intervention in 21.5% and valve adjustments in 37.3%, with no significant group differences. The valve adjustment resulted from abnormalities detected on MRI, not by the imaging procedure itself.

Follow-up MRI was more frequently needed in the tumor-related hydrocephalus group (17.4% vs. 4.5%; *p* = **0.014**), with differing clinical indications (*p* = **0.027**), though subsequent interventions were rare. Most patients were inpatients (75.3%), with a trend toward higher inpatient rates in the tumor-related hydrocephalus group (82.6% vs. 69.7%; *p* = 0.066). These subgroup differences highlight the adaptability of rMRI to a variety of clinical contexts.

The protocol was effective not only in detecting shunt-related complications, where ventricular size assessment is crucial, but also in evaluating tumor-related changes that inform long-term management. Importantly, all imaging was completed with minimal sedation (5%) and no significant technical failures, supporting the robustness of our protocol.

### 3.1. Comparison with Previous Studies

The study by Ramgopal et al. (2020) [22] demonstrated a significant reduction in head CT use (−21.5%) and a corresponding increase in rMRI use following implementation of rapid protocols in a pediatric emergency department. Although they reported a longer time to imaging and longer ED stay for rMRI compared to CT, the rate of missed pathology with rMRI was zero, while CT had a false-negative rate as high as 25% [22]. Our results demonstrate that rMRI is not only rapid (average scan time ~195 s) but also led to immediate clinical decisions in a significant number of cases, with only 1.9% requiring subsequent CT scans.

Boyle et al. (2014) compared rMRI and CT specifically for the evaluation of ventricular shunt malfunction, concluding that rMRI was not inferior to CT in diagnostic accuracy [7]. Our data further support this conclusion, with over half of our rMRI cases leading to actionable decisions, including surgical revisions and valve adjustments. Importantly, our imaging protocol enabled safe and reliable assessment of ventricular size and shunt function without exposing patients to ionizing radiation.

In terms of image quality, Brichtová et al. (2017) showed that turbo spin echo (TSE) sequences significantly reduce susceptibility artifacts in children with programmable shunt valves compared to single-shot techniques [9]. We achieved similar results using TSE and BLADE sequences, which provided excellent image quality even in the presence of metallic hardware. This supports the use of artifact-resistant protocols in neurosurgical emergencies, where diagnostic clarity is essential [9].

Lastly, Harbert et al. (2020) [3] examined a targeted CT reduction strategy for pediatric patients with hydrocephalus and TBI. While they succeeded in lowering CT use for hydrocephalus, they noted continued reliance on CT for trauma-related imaging [3]. Our study complements their findings by demonstrating that a well-defined, institution-wide rMRI protocol can successfully replace CT even in acute neurosurgical scenarios, leading to meaningful clinical outcomes without compromising diagnostic safety.

In summary, our findings reinforce the evolving role of rMRI as a frontline imaging strategy in pediatric neurosurgery. While limitations exist in trauma or hemorrhage-focused scenarios, rMRI offers substantial benefits for shunt evaluation, and ventricular assessment. Broader implementation of tailored rMRI protocols may enhance diagnostic accuracy while minimizing radiation exposure, sedation risks, and imaging delays.

### 3.2. Limitations

The findings of this study should be interpreted with caution due to its retrospective design and the relatively limited sample size. In addition, no healthy control group was included, as the study exclusively involved pediatric patients undergoing clinically indicated neuroimaging. Although our results support the use of rapid-sequence MRI in emergency pediatric neurosurgical scenarios, the conclusions may not be broadly applicable to all clinical settings, especially where MRI access or rapid protocols are not consistently available.

It is also important to acknowledge the focused diagnostic scope of our investigation, which primarily addressed ventricular size, tumor surveillance, and shunt evaluation. Other urgent neurological conditions—such as small intracranial hemorrhages, skull fractures, or diffuse axonal injuries—were not the primary focus and may require additional or alternative imaging sequences. Notably, our rMRI protocol did not routinely include sequences such as gradient echo (GRE), which are more sensitive for detecting hemorrhagic lesions. As such, rMRI should not be viewed as a universal substitute for CT in all emergency cases.

Furthermore, this study did not evaluate long-term clinical outcomes, including potential neurocognitive effects associated with the underlying neurological conditions or their treatment. These aspects, although well-documented in the literature, particularly for conditions like hydrocephalus or intraventricular tumors, were beyond the scope of our current analysis. Future prospective studies with larger cohorts and extended follow-up are needed to validate these findings and better define the role of rapid-sequence MRI in both acute and long-term pediatric neurosurgical care.

## 4. Conclusions

Rapid-sequence MRI (rMRI) is a fast, reliable, and radiation-free imaging modality for the emergency evaluation of pediatric patients with hydrocephalus, brain tumors, and CSF shunts. Our short protocol, completed in under 200 s, demonstrated high diagnostic value with minimal sedation requirements and a direct clinical impact in over one-third of cases, including surgical interventions and shunt adjustments. Subgroup analysis revealed significant diagnostic and management differences: patients in the tumor-related hydrocephalus group had more diverse drainage profiles and a higher need for follow-up MRI, reflecting their greater clinical complexity. Despite these differences, rMRI proved equally effective across all subgroups, supporting its flexibility and broad applicability.

Overall, rMRI offers a practical alternative to cranial CT in most pediatric neurosurgical emergencies, combining diagnostic accuracy with improved patient safety. Further prospective studies are warranted to refine pathology-specific protocols and assess long-term clinical outcomes.

## Figures and Tables

**Table 1:** Patient characteristics.

Variable	Cases (%)
Total Number of Cases	158 (100)
Gender Distribution:	
Male	110 (69.6)
Female	48 (30.4)
Age * in years [Range]	7.09 ± 4.63 [0.1–21.5]
Follow-up Duration * in months	21.92 ± 29.35
Study Groups:	
Tumor non-related hydrocephalus	89 (56.3)
Tumor-related hydrocephalus	69 (43.7)
Drainage Categories:	
Internal	146 (92.4)
External	5 (3.2)
No CSF drainage	7 (4.4)
Diagnose:	
Congenital/Post-hemorrhagic hydrocephalus	58 (36.7)
Pilocytic Astrocytoma	23 (14.6)
Medulloblastoma	17 (10.8)
Idiopathic Aqueduct Stenosis	10 (6.3)
Others **	50 (31.7)
Indications for Rapid MRI (rMRI):	
Ventricular size monitoring	152 (96.2)
Exclusion of increased intracranial pressure (ICP)	2 (1.3)
Exclusion of hemorrhage	1 (0.6)
Postoperative follow-up	1 (0.6)
Shunt follow-up Fluid in the mastoid cells	1 (0.6) 1 (0.6)
Patient Type:	
Inpatients	119 (75.3)
Outpatients	39 (24.7)
Consequences following rMRI:	83 (52.5)
Shunt valve adjustments	59 (37.3)
Surgical intervention	34 (21.5)
Need for Sedation during rMRI	8 (5.1)
Additional Imaging:	
Need for regular MRI after rMRI	16 (10.1)
Need for regular cranial CT-Scan after rMRI	3 (1.9)

(%) Data in parenthesis are percentages. * Data are given as mean ± standard deviation. ** Others: Diffuse glioma, Gangliocytoma, Ependymoma, Neuroblastoma, Diffuse leptomeningeal glioneuronal tumor, Teratoid/rhabdoid tumor, Vein of Galen malformation, Germinoma, Pineoblastoma, Postinfectious hydrocephalus, Pontine glioma, Sinus vein thrombosis, Arachnoid cyst, Dandy–Walker malformation, Hydranencephaly, Embryonal tumor with multilayered rosettes, Meningioma, Neuroblastoma, Thalamic glioma.

**Table 2:** Statistical comparison between the tumor non-related and tumor-related hydrocephalus groups.

	Tumor Non-Related Hydrocephalus	Tumor-Related Hydrocephalus	*p*-Value *
**Cases**	89 (56.3)	69 (43.7)	NS
Age (Mean) in years *	6.3 ± 4.4	8.1 ± 4.7	**0.010**
Gender:			
Male	57 (64.0)	53 (76.8)	0.116
Female	32 (36.0)	16 (23.2)	
Diagnose:			
Post-hemorrhagic hydrocephalus	49 (55.1)	0 (0.0)	**<0.001**
Pilocytic Astrocytoma (WHO I)	0 (0.0)	23 (33.3)	
Medulloblastoma	0 (0.0)	17 (24.6)	
Idiopathic aqueduct stenosis	10 (11.2)	0 (0.0)	
Congenital hydrocephalus	9 (10.1)	0 (0.0)	
Others **	21 (23.6)	29 (42.0)	
Drainage Categories:			
Internal	89 (100)	57 (82.6)	**<0.001**
External	0 (0)	5 (7.2)	
No CSF drainage	0 (0)	7 (10.1)	
Indication rapid seq. MRI (rMRI)			
Ventricular size	88 (98.9)	64 (92.8)	0.259
Exclusion of bleeding	0 (0.0)	1 (1.4)	
Exclusion of increased ICP	1 (1.1)	1 (1.4)	
Postoperative course	0 (0.0)	1 (1.4)	
Shunt function/course	0 (0.0)	1 (1.4)	
Fluid in mastoid cell	0 (0.0)	1 (1.4)	
Patient’s Presentation Type:			
Inpatients	62 (69.7)	57 (82.6)	
Outpatients	27 (30.3)	12 (17.4)	0.066
Consequences following rMRI:	50 (56.2)	33 (47.8)	0.337
Shunt valve adjustments	38 (42.7)	21 (30.4)	0.137
Surgical intervention	17 (19.1)	17 (24.6)	0.439
Need for Sedation during rMRI	4 (4.5)	4 (5.8)	0.730
Additional Imaging:			
Need for regular MRI after rMRI	4 (4.5)	12 (17.4)	**0.014**
Need for regular cranial CT-Scan after rMRI	1 (1.1)	1 (1.4)	0.581
Indication for reg. MRT:			
Ventricular size	3 (3.4)	8 (11.6)	
Post-OP follow-up	0 (0.0)	1 (1.4)	**0.027**
Staging	0 (0.0)	1 (1.4)	
Follow-up	0 (0.0)	1 (1.4)	
Infarct exclusion	1 (1.1)	0 (0.0)	
Preoperative	0 (0.0)	1 (1.4)	
Consequence of regular MRI:			
EVD removal	0 (0.0)	1 (1.4)	0.251
Shunt adjustment	3 (3.4)	1 (1.4)	
Operative Shunt-Revision	0 (0.0)	1 (1.4)	
Preoperative	0 (0.0)	1 (1.4)	
Surgical placement of a VP shunt	0 (0.0)	1 (1.4)	

(%) Data in parenthesis are percentages. * Data are given as mean ± standard deviation. ** Others: Diffuse glioma, Gangliocytoma, Ependymoma, Neuroblastoma, Diffuse leptomeningeal glioneuronal tumor, Teratoid/rhabdoid tumor, Vein of Galen malformation, Germinoma, Pineoblastoma, Postinfectious hydrocephalus, Pontine glioma, Sinus vein thrombosis, Arachnoid cyst, Dandy–Walker malformation, Hydranencephaly, Embryonal tumor with multilayered rosettes, Meningioma, Neuroblastoma, Thalamic glioma.

## Data Availability

The data presented in this study are available upon request from the corresponding author.

## References

[1] Harbert A., Bradford K., Ritter V., Northam W.T., Quinsey C. (2020). National imaging trends in pediatric traumatic brain injury and hydrocephalus. World Neurosurg..

[2] George E., Barkovich M.J. (2026). Pediatric Neuroimaging. Swaiman’s Pediatric Neurology.

[3] Harbert A., Northam W., Elton S., Quinsey C. (2020). Targeted head CT reduction for pediatric patients with hydrocephalus and traumatic brain injury: Academic center institutional experience as an example of opportunities for further improvement. Child’s Nerv. Syst..

[4] Hauptmann M., Byrnes G., Cardis E., Bernier M.-O., Blettner M., Dabin J., Engels H., Istad T.S., Johansen C., Kaijser M. (2023). Brain cancer after radiation exposure from CT examinations of children and young adults: Results from the EPI-CT cohort study. Lancet Oncol..

[5] Smith-Bindman R., Chu P.W., Firdaus H.A., Stewart C., Malekhedayat M., Alber S., Bolch W.E., Mahendra M., de González A.B., Miglioretti D.L. (2025). Projected lifetime cancer risks from current computed tomography imaging. JAMA Intern. Med..

[6] Wylie M.C., Merritt C., Clark M., Garro A.C., Rutman M.S. (2014). Imaging of pediatric head injury in the emergency department. Pediatr. Emerg. Care.

[7] Boyle T.P., Paldino M.J., Kimia A.A., Fitz B.M., Madsen J.R., Monuteaux M.C., Nigrovic L.E. (2014). Comparison of rapid cranial MRI to CT for ventricular shunt malfunction. Pediatrics.

[8] Mehta H., Acharya J., Mohan A., Tobias M., LeCompte L., Jeevan D. (2016). Minimizing radiation exposure in evaluation of pediatric head trauma: Use of rapid MR imaging. Am. J. Neuroradiol..

[9] Brichtová E., Šenkyřík J. (2017). SSh versus TSE sequence protocol in rapid MR examination of pediatric patients with programmable drainage system. Child’s Nerv. Syst..

[10] Cohen A.R., Caruso P., Duhaime A.-C., Klig J.E. (2015). Feasibility of “rapid” magnetic resonance imaging in pediatric acute head injury. Am. J. Emerg. Med..

[11] Czech T., Pardo A.C. (2020). Utility of rapid sequence magnetic resonance imaging in guiding management of patients with neonatal seizures. Pediatr. Neurol..

[12] Kabakus I.M., Spampinato M.V., Knipfing M., Cervantes G., Patel A., Eskandari R., Yazdani M. (2021). Fast brain magnetic resonance imaging with half-Fourier acquisition with single-shot turbo spin echo sequence in detection of intracranial hemorrhage and skull fracture in general pediatric patients: Preliminary results. Pediatr. Emerg. Care.

[13] Ryan M.E., Jaju A., Ciolino J.D., Alden T. (2016). Rapid MRI evaluation of acute intracranial hemorrhage in pediatric head trauma. Neuroradiology.

[14] Sheridan D.C., Newgard C.D., Selden N.R., Jafri M.A., Hansen M.L. (2017). QuickBrain MRI for the detection of acute pediatric traumatic brain injury. J. Neurosurg. Pediatr..

[15] De Jong G., Kannikeswaran N., DeLaroche A., Farooqi A., Sivaswamy L. (2020). Rapid sequence MRI protocol in the evaluation of pediatric brain attacks. Pediatr. Neurol..

[16] Flom L., Fromkin J., Panigrahy A., Tyler-Kabara E., Berger R.P. (2016). Development of a screening MRI for infants at risk for abusive head trauma. Pediatr. Radiol..

[17] Smith-Bindman R., Alber S.A., Kwan M.L., Pequeno P., Bolch W.E., Bowles E.J., Greenlee R.T., Stout N.K., Weinmann S., Moy L.M. (2025). Medical imaging and pediatric and adolescent hematologic cancer risk. N. Engl. J. Med..

[18] Niederhauser B., McDonald R., Eckel L., Keating G., Broomall E., Wetjen N., Diehn F., Schwartz K., Hunt C., Welker K. (2013). Retrospective review of rapid pediatric brain MR imaging at an academic institution including practice trends and factors affecting scan times. Am. J. Neuroradiol..

[19] Ashley W.W., McKinstry R.C., Leonard J.R., Smyth M.D., Lee B.C., Park T.S. (2005). Use of rapid-sequence magnetic resonance imaging for evaluation of hydrocephalus in children. J. Neurosurg. Pediatr..

[20] Thompson E.M., Baird L.C., Selden N.R. (2014). Results of a North American survey of rapid-sequence MRI utilization to evaluate cerebral ventricles in children. J. Neurosurg. Pediatr..

[21] O’Neill B.R., Pruthi S., Bains H., Robison R., Weir K., Ojemann J., Ellenbogen R., Avellino A., Browd S.R. (2013). Rapid sequence magnetic resonance imaging in the assessment of children with hydrocephalus. World Neurosurg..

[22] Ramgopal S., Karim S.A., Subramanian S., Furtado A.D., Marin J.R. (2020). Rapid brain MRI protocols reduce head computerized tomography use in the pediatric emergency department. BMC Pediatr..

